# Increased SNAT1 is a marker of human osteosarcoma and potential therapeutic target

**DOI:** 10.18632/oncotarget.20693

**Published:** 2017-09-05

**Authors:** Miaomiao Wang, Ying Liu, Wenzheng Fang, Ke Liu, Xiaodong Jiao, Zhan Wang, Jiejun Wang, Yuan-Sheng Zang

**Affiliations:** ^1^ Department of Medical Oncology, Changzheng Hospital, Shanghai 200070, China

**Keywords:** SNAT1, osteosarcoma, prognosis, metastasis, MMP9

## Abstract

**Background:**

SLC38A1/SNAT1 has been found to play an essential role in human development, but its role in osteosarcoma (OS) has yet to be evaluated. The purpose of this study was to assess the expression of SLC38A1/SNAT1 in patients with OS, and further investigate the mechanisms by which it affects tumor growth and metastasis.

**Methods:**

Tissue microarray blocks and immunohistochemical studies were carried out to assess the expression of SNAT1 in 165 OS specimens. Its correlation with clinicopathological characteristics was then analyzed. The function of SNAT1 in OS cells was investigated by silencing SNAT1 using SNAT1-shRNA *in vitro* and *in vivo*.

**Results:**

SNAT1 was highly expressed in 85% OS and significantly closely associated with pulmonary metastasis. Patients with high SNAT1 expression survived for shorter periods than those with low SNAT1 expression. Suppression of endogenous SNAT1 led to inhibition of cell proliferation, cell colony formation, and cell migration *in vitro*, and retarded tumor growth in xenograft models. Silencing SNAT1 reduced expression of MMP9, vimentin, fibronectin, p-Akt, p-mTOR, and VEGF.

**Conclusions:**

Our results indicated that increased expression of SNAT1 is a common event in OS. SNAT1 played an essential role in the development and progression of osteosarcoma, which may serve as a prognostic and therapeutic marker of OS.

## INTRODUCTION

Osteosarcoma (OS) is the most common primary malignant bone tumor in adolescents. It has a high metastatic rate and poor prognosis [1, 2]. Multi-agent chemotherapy increases the 5-year overall survival rate of patients with localized disease, which then ranges from 60% to 70%. However, the 5-year survival for those with pulmonary metastasis is only 11% to 20% [3–5]. Therefore, it is very important to identify potential therapeutic targets suitable for the treatment of patients with OS. Previous studies have confirmed that certain abnormal expression of proteins in pulmonary metastasis of OS may initiate tumor cells proliferation and metastasis [6]. In addition, intracellular protein imbalances lead to the proliferation of tumor cells directly through the activation of protein kinase pathways [7].

Amino acid transporters play an important role in various cell life activities, including energy metabolism, detoxication, neutron transmission, and even malignant transformation of mammal cell [8–10]. Among these amino acid transporters, system A has been found to be overexpressed in various human solid cancers, includinghepatocellular carcinoma [11], hilar cholangiocarcinoma [12], and breast cancer [13]. System A amino acid transporter is a Na+-dependent active transport system known to mediate the uptake of amino acids with small side chains (e.g, alanine, serine, proline, and glutamine) [10]. Its activity is heavily influenced by pH, cell volume, and a variety of hormones, such as insulin, glucagon, and insulin-like growth factor-I. There are 3 System A amino acid transporters: SNAT1, SNAT2, and SNAT4 (previously referred to as ATA1/SLC38A1, ATA2/SLC38A2, and ATA3/SLC38A4, respectively), all encoded by the SLC38 gene family [14]. Among these 3 transporters, silencing endogenous SNAT1 inhibited cell proliferation of various tumor cells [11, 13]. Moreover, SNAT1 overexpression was significantly closely correlated with tumor recurrence and poor outcome in patients with hilar cholangiocarcinoma [12]. However, the mechanisms underlying SNAT1’s promotion of the development and progression of OS remain unclear.

In order to elucidate the relationship between SNAT1 and OS, we assessed the expression of SNAT1 in 165 patients with OS, and then investigated its biological effect on OS cells *in vitro* and *in vivo*. Our data revealed that SNAT1 is highly expressed in patient tissues with OS, and plays a suppressive role in the development and progression of OS. These findings shed a new light on the biological functions of SNAT1 on OS, which may provide a suitable prognostic and therapeutic target for OS.

## RESULTS

### Expression of SNAT1 in OS and its correlation with clinicopathological characteristics of OS patients

**Figure 1:**
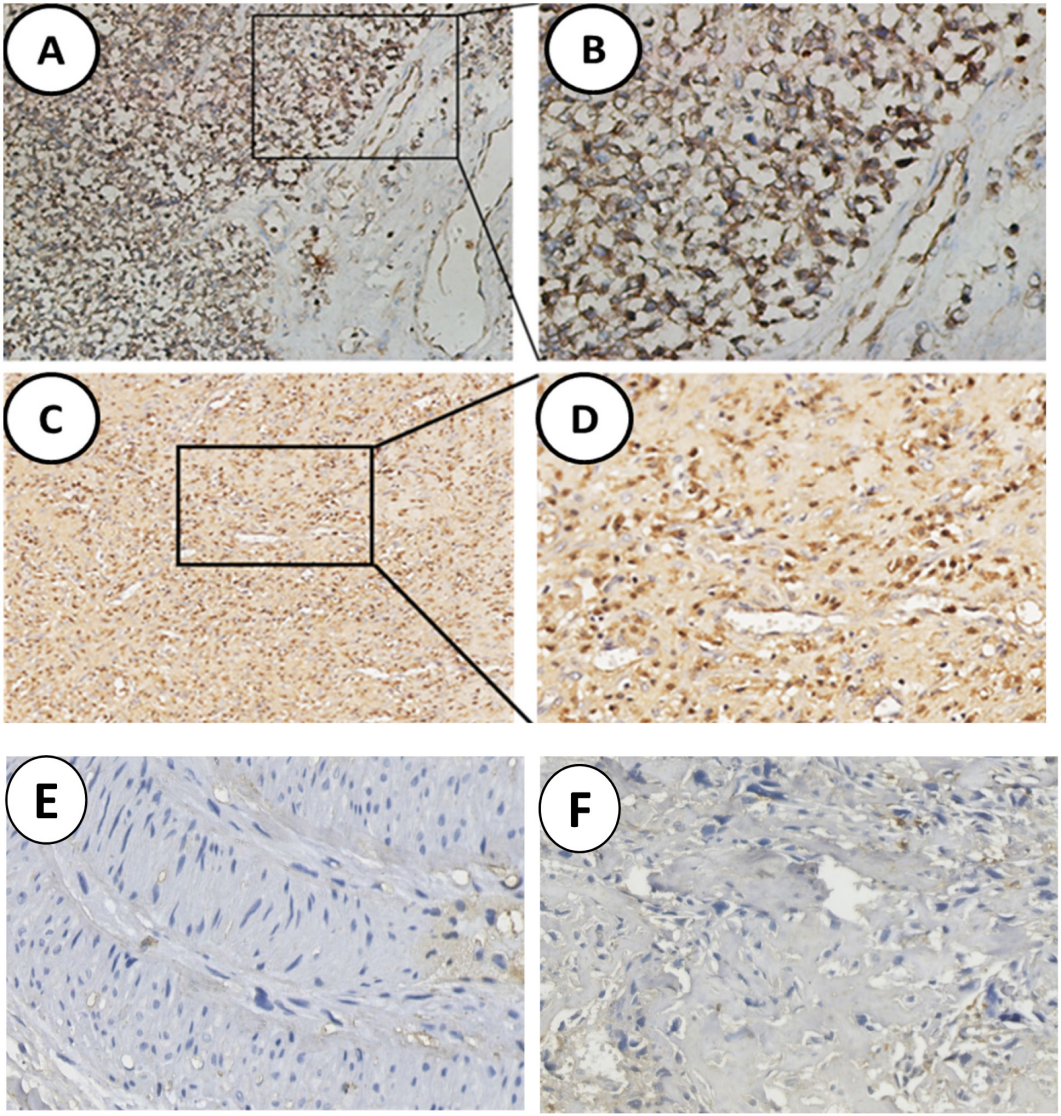
Analysis of SNAT1 expression in human osteosarcoma **(A, C)** High level of SNAT1 expression in tumor cells; **(B, D)** Enlargement of tissues in the frames from A, C, respectively. **(E, F)** Noncancerous tissues showed negative staining of SNAT1. Original magnification of A, C: 100×; Original magnification of B, D-F: 200×.

As shown in Figure 1, SNAT1 was mainly localized in cytoplasm, and its expression was markedly higher in osteosarcoma cells (Figure 1A–1D) than in adjacent tissues (Figure 1E–1F). According to SNAT1 expression in OS, 165 patients were divided into a high SNAT1 expression group (N=111) and low SNAT1 expression group (N=54). Table 1 shows the correlation between SNAT1 expression and clinicopathological parameters of osteosarcoma. No significant relationship was found between SNAT1 expression and tumor size, tumor location, gender, or age. However, high levels of SNAT1 expression were found to be significantly closely associated with survival time (<3y *vs.* ≥3y: 82.1% *vs.* 33.3%, *P*<0.001). As for metastatic status, SNAT1 expression occurred more frequently in lung invasion tumors (83.3%) than tumors without invasion (0%, *P* < 0.001).

**Table 1:** Association between high SNAT1 expression and clinicopathological factors in osteosarcoma

Clinicopathological factor	N	SNAT1		χ2	*P*
		Low (%)	High (%)		
**Size**					
>8cm	101	19(18.8)	83(82.2)	25.12	0.001
≤8cm	64	36(56.2)	28(43.8)		
**Location**					
Femora	62	17(27.4)	45(72.6)	5.02	*NS*
Tibia	54	24(44.4)	30(55.5)		
Others	49	13(26.5)	36(73.4)		
**Survival time**					
<3y	116	21(17.9)	95(82.1)	37.94	0.001
≥3y	49	33(66.7)	16(33.3)		
**Metastasis status**					
Lung	99	17(16.7)	82(83.3)	62.49	0.001
Others	41	12(30)	29(70)		
None	25	25(100)	0(0)		
**Sex**					
Male	118	36(31.2)	82(68.8)	0.72	*NS*
Female	47	18(37.5)	29(62.5)		
**Age, y**					
<18	124	40(33.3)	82(66.7)	0.17	*NS*
≥18	41	12(30)	29(70)		

### SNAT1 expression and survival time in patients with OS

**Figure 2:**
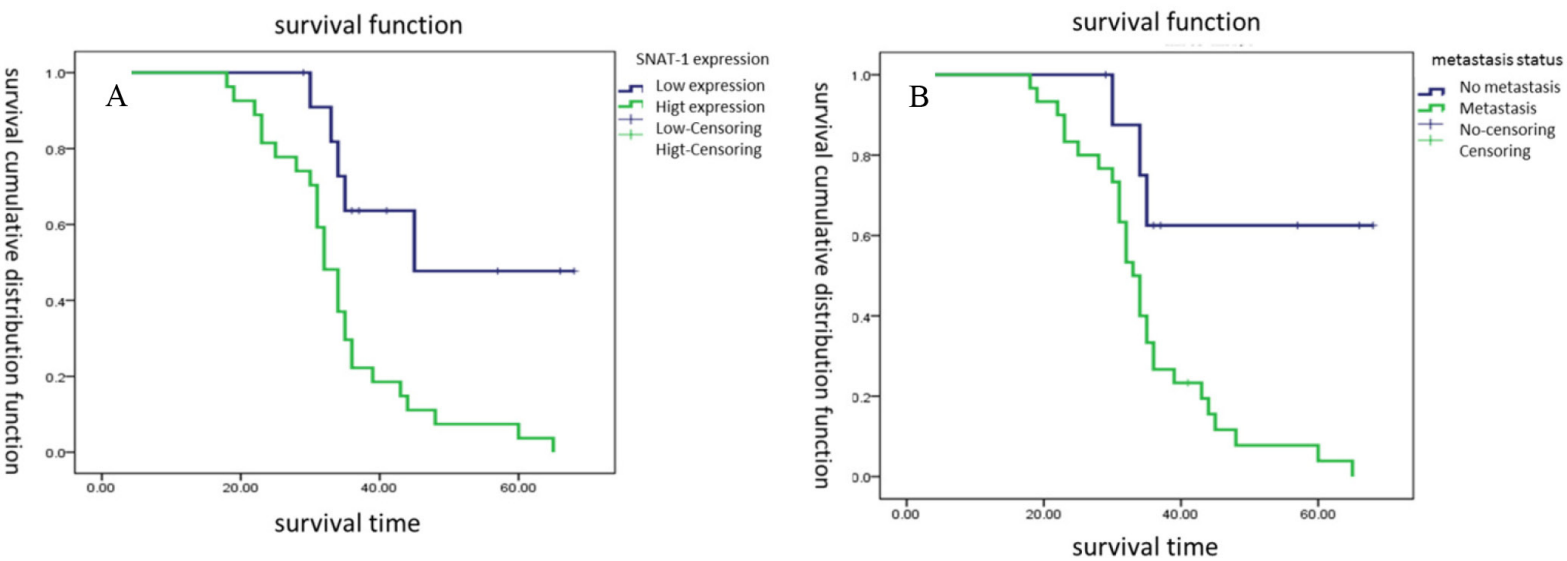
Survival cumulative distribution function of SNAT1 in patients with osteosarcoma **(A)** Survival durations were significantly worse in patients with high expression of SNAT1 than in those with low expression of SNAT1. **(B)** Patients with metastatic tumors had a shorter survival duration than those without metastatic tumors.

This cohort consisted of 118 male and 47 female patients with a median age of 18 years, ranging from 12 to 60 years. The median cumulative survival duration in these patients with resected osteosarcoma was 36 months. Kaplan-Meier survival analyses revealed that the patients with SNAT1 overexpression tumors had significantly shorter median survival duration than patients without such tumors (*P* < 0.001) (Figure 2A). Significant differences in the median duration of survival were observed between patients with metastatic tumors and those without metastatic tumors (*P* <0.001) (Figure 2B).

### Knockdown of SNAT1 and OS cell growth

**Figure 3:**
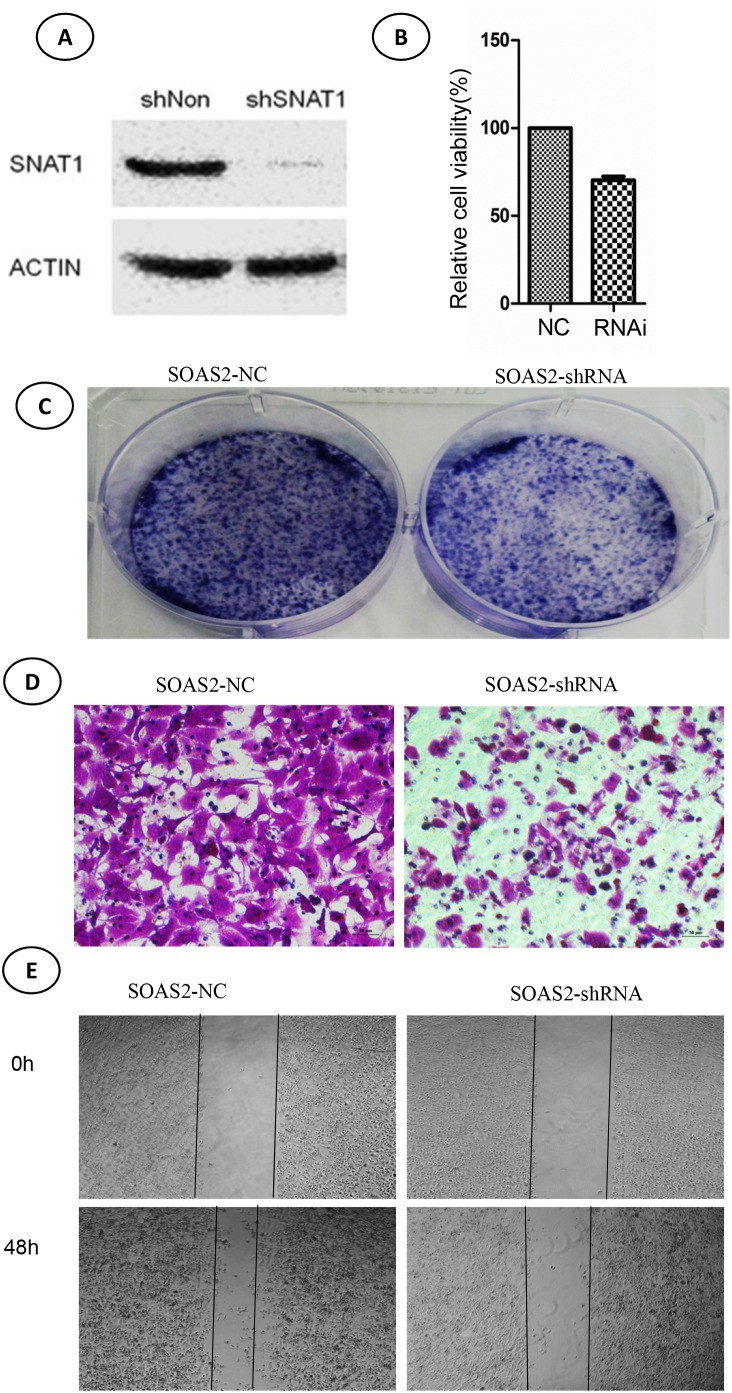
Effects of SNAT-shRNA on cell viability, colony formation, and migration in soas-2 cell lines **(A)** Western blotting showed expression of SNAT1 after transfection with SNAT1-shRNA and control for 48 hours. **(B)** Cell viability was determined by CCK8 assay. **(C)** Cell viability was determined by colony formation assay. **(D, E)** Cell migration was determined by transwell assay (D) and wound healing assay (E).

Next, we investigated the biological mechanism underlying the role of SNAT1 in the progression of osteosarcoma. SOAS-2 cells that stably expressed sh-SNAT1 RNA were firstly established. As shown in Figure 3A, SNAT1-shRNA transfection results in a sharp reduction in SNAT1 expression in OS cells. In addition, cell viability (Figure 3B) and colony formation (Figure 3C) were markedly inhibited by SNAT1-shRNA.

### Knockdown of SNAT1 repressed the ability of invasion of OS

**Figure 4:**
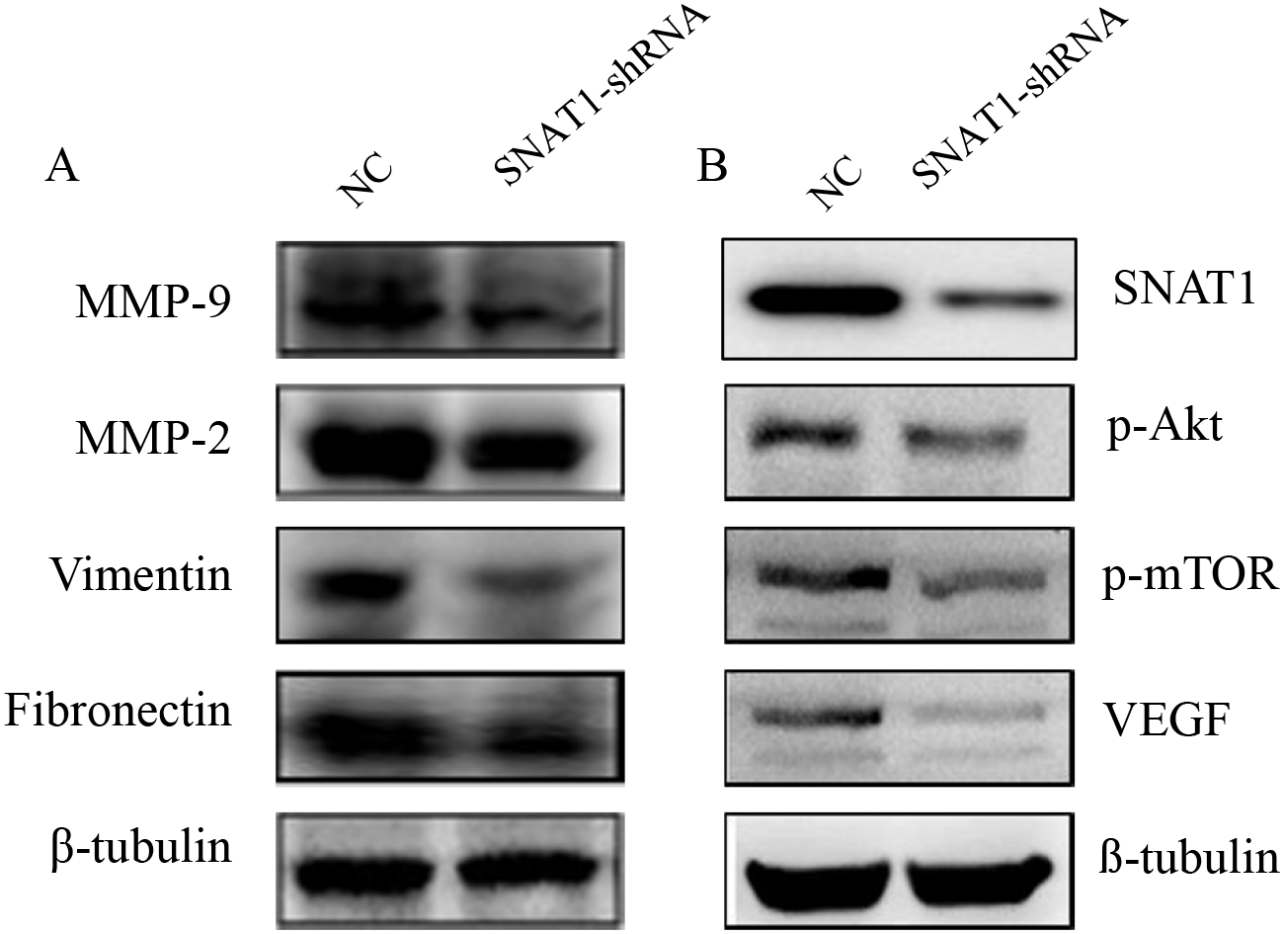
After silencing SNAT1 using SNAT1-shRNA, western blot assay was used to detect the expression of MMP-9, MMP-2, Vimentin, and Fibronectin (A), as well as VEGF, p-mTOR, and p-Akt (B)

Moreover, we detected the effect of SNAT1 inhibition on cell migration by wound healing assay (Figure 3D) and transwell assay (Figure 3E), and found cell migration to be significantly inhibited when SNAT1 activity was inhibited. In line with this, we found that expression of other metastasis-related proteins, such as MMP9, MMP2, vimentin, and fibronectin, was also lower in SNAT1 knockdown cells than in cells transfected with shRNA empty vector (Figure 4A).

### Knockdown of SNAT1 Inhibited the Akt/mTOR pathway and VEGF

In the metastatic progress of OS, the PI3K/Akt/mTOR pathway is often aberrantly activated. The activated PI3K/Akt/mTOR pathway contributes to cellular transformation via downstream effectors such as VEGF [15]. We then detected the effects of SNAT1 on VEGF, p-mTOR, and p-Akt expression. After transfection with SNAT1-shRNA in SOAS-2 cells, a significantly reduction in p-Akt, p-mTOR, and VEGF levels was observed (Figure 4B). Taken together, these results suggested that the inhibitory effects of SNAT1 on the maintenance of osteosarcoma cell metastasis occurred mainly through blockage of Akt phosphorylation.

### SNAT1 accelerated OS growth *in vivo*

**Figure 5:**
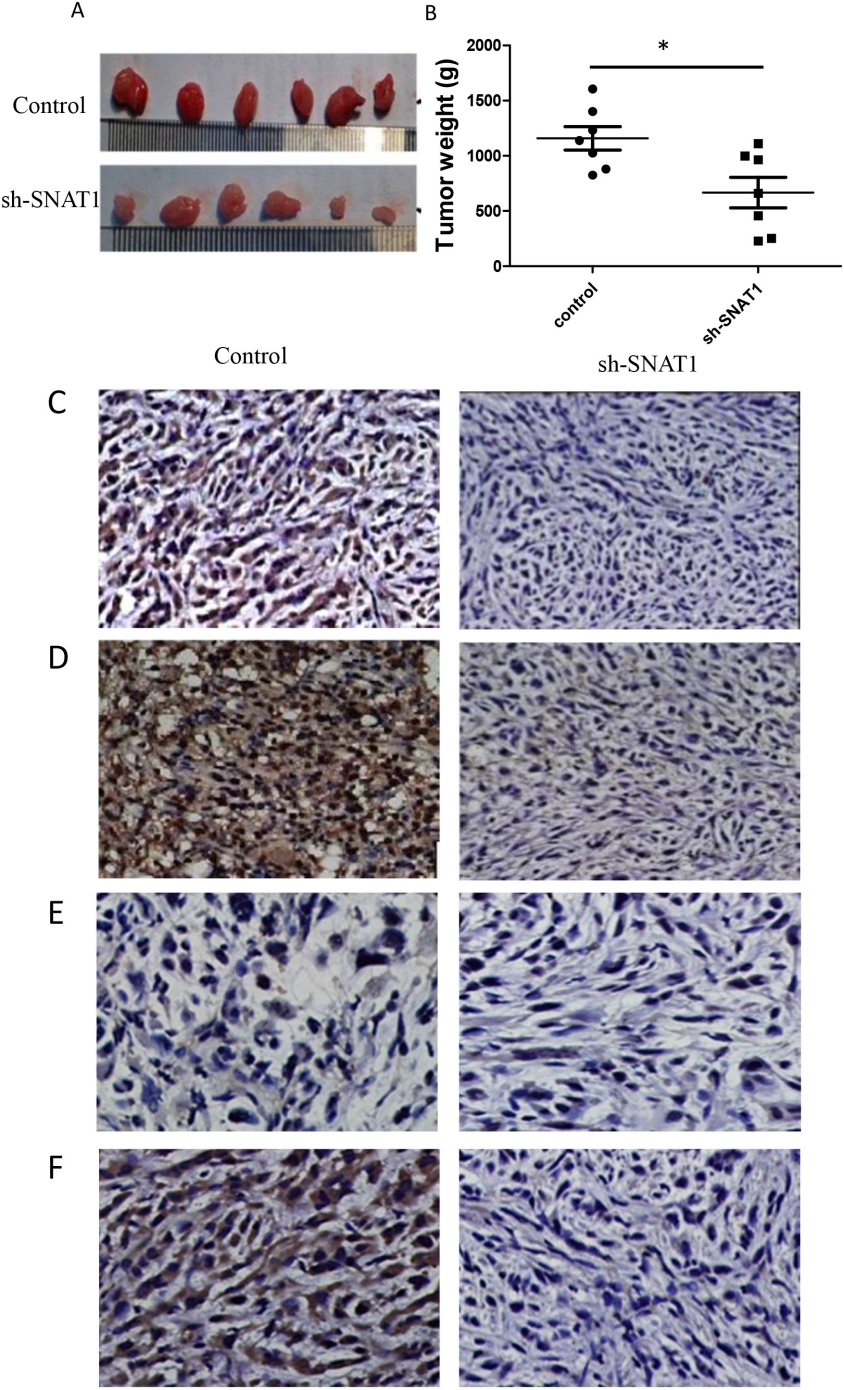
Silencing SNAT1 inhibited tumor growth *in vivo* **(A)** Representative images of resected tumors. SOAS-2 cells stably expressing sh-SNAT1 or negative control (shNon) were subcutaneous injected into the capsule of the right armpit of nude mice (n = 6/group). After 18d of subcutaneous injected, the mice were anesthetized, and the tissue of Osteosarcoma were collected and tumor weights were calculated **(B)**. Tumors from the mice were stained for MMP9 **(C)**, VEGF **(D)**, p-Akt **(E)**, and p-mTOR **(F)**.

To determine the functional significance of expression of SNAT1 in OS growth *in vivo*, SOAS2 cells stably expressing SNAT1-shRNA or the vehicle control were subcutaneously transplanted into the capsule of the right armpits of nude mice. After 18 days, mice were killed and the tumors were collected. Knockdown of SNAT1 caused significant retardation of tumor growth *in vivo* relative to the group treated with vehicle control (Figure 5A–5B). Furthermore, the protein expression levels of MMP-9, p-Akt, p-mTOR, and VEGF were significantly lower in the SNAT1-shRNA group than in the control group (Figure 5C–5F). Both *in vivo* and *in vitro* data suggest that SNAT1 represses OS cell growth and invasion, which is most likely to take place through inhibition of the PI3K/Akt/mTOR pathway.

## DISCUSSION

Of all the primary bone cancers, osteosarcoma is the most common in adolescents, and it has considerably poor prognosis and a pronounced ability to metastasize to the lung [16]. Glutamine transporters are conditionally essential in cancer cells, being utilized to generate energy for anabolic purposes, and are potential targets for cancer therapy [17, 18]. SNAT1 is a subtype of the amino acid transporter that has been investigated most intensively in human solid cancers [11–13], yet its roles in OS remain unclear. In the present study, we found SNAT1 to be highly expressed in osteosarcoma tissues, which was significantly closely associated with pulmonary metastasis and the outcome of patients with OS. Moreover, SNAT1 inhibition *in vitro* was found to reduce OS cell growth and metastasis. These data collectively suggested that SNAT1 activation in patients with OS may play a critical role in cancer development and progression.

Osteosarcoma is characterized by a high rate of metastasis with a mean 5-year survival rate of <20% [1]. To reduce mortality and morbidity in OS patients, an extensive knowledge of the mechanism underlying osteosarcoma metastasis is required. In the current study, we established that SANT1 contributes to metastatic progression of osteosarcoma. By immunohistochemistry, we found SNAT1 to be highly expressed in osteosarcoma tissues. We also found SNAT1 expression to be correlated with metastatic status. We then performed a knockdown assay to evaluate the effect of SNAT1 on metastatic potential of osteosarcoma cells *in vitro*. In line with our data from immunohistochemical analysis, we found metastatic behavior of osteosarcoma cells (SOAS-2 cells) to be markedly inhibited by silencing SANT1 both *in vitro* and *in vivo*. Based on these data, we concluded that SNAT1 plays important roles in metastatic progression of osteosarcoma.

The PI3K/Akt/mTOR pathway is frequently activated in OS and contributes to disease initiation and development, including tumorigenesis, proliferation, invasion, and cell cycle progression [19–21]. Inhibition of this pathway through small-molecule compounds represents an attractive therapeutic approach to the treatment of a variety of human tumors [22]. Glutamine, the supply of which is instrumental in tumor growth, is a preferred SNAT1 substrate. Overexpression of SNAT1 can lead to increased Gln transport to the cells and promote of glutaminolysis [23]. It thus appears likely that SNAT1 knockout causes Gln deprivation, which in turn limits the PI3K/Akt/VEGF pathway, a sequence of events that has been reported in carcinoma cells [24]. In addition, glutaminolysis activation appears to be promoted by mTOR [25]. Not surprisingly, we found that the phosphorylation levels of mTOR and Akt to be markedly reduced by silencing SNAT1. Angiogenesis is also a crucial step in tumor formation and progression [26], and several recent studies support the notion that inhibition of mTOR is associated with decreased VEGF expression [27, 28]. Consistently, our data showed that SNAT1 inhibition also leads to significant reductions in VEGF expression. These findings suggest that the function of SNAT1 in OS metastasis may involve in the activation of the above pathway, further promoting angiogenesis. Establishing the effect of SNAT1 on the PI3K/mTOR and VEGF pathways is only one initial step toward resolving the mechanism. This phenomenon should be examined in more OS cells and in different types of tumor cells in further studies.

To the best of our knowledge, this is the first study to systemically investigate the expression pattern and cellular mechanism associated with SANT1 in OS patients. Our data provide evidence that SNAT1 expression was increased in osteosarcoma tissues, and it was associated with survival time and metastasis status. Moreover, both *in vitro* and *in vivo* studies revealed that knockdown of SNAT1 could suppress OS growth and metastasis, which probably involves blocking the PI3K/Akt/mTOR pathway. Our data collectively showed that SNAT1 may be involved in the development and progression of osteosarcoma, which could be explored as a novel and efficient prognostic marker for patients with OS.

## MATERIALS AND METHODS

### Patient specimens and tissue microarray construction

A total of 165 previously untreated patients with OS who underwent curative surgery at Changzheng Hospital or Changhai Hospital from 2006 to 2011 were enrolled. Patients with other sarcomas, such as Ewing’s sarcoma, chondrosarcoma, and osteochondroma, were excluded from this study. The patients’ medical records including age at diagnosis, gender, tumor location, tumor size (diameter), metastasis status, and survival time were obtained. The mean age of patients at tumor resection was 18 years (male vs. female=118:47). Clinical follow-up was available with mean follow-up lasting 37 months, ranging from 21–73 months. Four paraffin-embedded tissue microarray blocks of osteosarcoma and para-carcinoma tissues obtained from these patients were created using a manual arrayer (Beecher Instruments) as described previously [29]. Each block had 2 1.5 mm cores of primary tumor tissue and at least 1 1.5 mm core of matched non-neoplastic tissue. For patients with lung metastasis, 1 or 2 1.5 mm cores of metastatic tissue were included. The detailed information is listed in Table 1. Written informed-consent documents were obtained from all participants before this study and the use of the human specimens was approved by the Changzheng/Changhai Hospital Institutional Review Board.

### Immunostaining

Consecutive sections (4 μm) of paraffin-embedded tissue microarrays blocks were prepared and processed for immunohistochemical analysis. The expression of SNAT1, p-mTOR, p-Akt, and VEGF proteins in the sections and in animal models was detected with appropriate antibodies against p-mTOR (dilution, 1:50; clone Y391; Abcam), SNAT1 (dilution, 1:100; clone S104-32; Abcam), p-Akt (dilution, 1:100; clone S473; Abcam), and VEGF (dilution, 1:200; clone SP28; Lab Vision). Two individuals evaluated the expression of these proteins using an Olympus CX31 microscope (Olympus Optical). A semiquantitative scoring system was used [30]. Specifically, underexpression was defined as no staining of tumor tissue or less positive staining than in matched normal tissue, normal expression was defined as positive staining positivity similar to that of matched normal tissue, and high expression was defined as significantly more positive staining than in normal tissue. Normal and negative staining were here defined as low SNAT1 expression. Staining was scored independently by 2 individuals who were blinded to each other’s findings. All conflicting calls on scoring were adjudicated by a third individual.

### Plasmids and transfections

The shRNA-SNAT1 and unspecific scrambled shRNA plasmids were purchased from Genechem Company, Shanghai, China. At 24 h before transfection, 1×10^5^ cells were seeded in 6-well plates. Transfection of shRNA was carried out using Lipofectamine™ 2000 reagent (Invitrogen, Karlsruhe, Germany) with 4 μg hRNA plasmid per the manufacturer’s instructions.

### Cell lines and culture conditions

The human osteosarcoma cell line SOAS-2 was purchased from the American Type Culture Collection (ATCC). The cell lines were cultured in a 37°C humidified atmosphere containing 95% air and 5% CO_2_.

### Western-blot analysis

After transfection, osteosarcoma cell lines were prepared for Western blot analyses. Standard Western blotting was performed using a rabbit antibody against human SNAT1, MMP9, MMP2, vimentin, fibronectin, VEGF, p-mTOR, p-Akt, and an anti-rabbit IgG antibody, which was a horseradish peroxidaselinked F(ab′)2 fragments obtained from a donkey (Amersham). Equal protein sample loading was monitored by probing the same membrane filter with an anti-β-tubulin or actin antibody.

### Cell proliferation assay

At 12 h after transfection, cells were digested and 5000 cells were seeded in 96-well plates, and incubated in medium with 10% FBS. At 24 h, 48 h, and 72 h, CCK8 assay (Dojindo Kumamoto, Japan) was performed to measure the final results. The experiment was repeated three times.

### Colony formation assay

At 24 h after transfection, cells were digested and seeded in 6-well plates in triplicate at a density of 500 cells/well for 14 days at 37°C. The colonies were fixed with methanol/acetone (1:1) and stained with crystal violet. Colonies with more than 50 cells per colony were counted.

### Xenograft models

The nude mice were purchased from the animal experiment center of the Second Military Medical University. The growth rates of those tumor cell lines in nude mice were determined as described previously [31].

### Statistical analysis

Categorical data were analyzed using χ^2^ statistical testing. Within-group correlations of continuous and ordinal variables were assessed using Pearson’s correlation coefficient or Spearman’s rank correlation coefficient when appropriate. The Kaplan-Meier method was used to estimate survival rates, and the log-rank test was used to assess survival differences between groups. Analyses were performed using SPSS statistical software. The significance of the *in vitro* data was determined using Student’s *t* test (two-tailed). For all tests, a *P* < 0.05 was considered as statistically significant.

## Abbreviations

OS: Osteosarcoma
